# Long-term effectiveness of a lifestyle intervention on the prevention of type 2 diabetes in a middle-income country

**DOI:** 10.1038/s41598-020-71119-2

**Published:** 2020-08-25

**Authors:** Mojtaba Lotfaliany, Mohamad Ali Mansournia, Fereidoun Azizi, Farzad Hadaegh, Neda Zafari, Arash Ghanbarian, Parvin Mirmiran, Brian Oldenburg, Davood Khalili

**Affiliations:** 1 Non-Communicable Disease Control, School of Population and Global Health, University of Melbourne, Parkville, VIC 3010 Australia; 2 Prevention of Metabolic Disorders Research Center, Research Institute for Endocrine Sciences, Shahid Beheshti University of Medical Sciences, P O Box: 19395-4763, Tehran, Iran; 3 Department of Epidemiology and Biostatistics, School of Public Health, Tehran University of Medical Sciences, Tehran, Iran; 4 Endocrine Research Center, Research Institute for Endocrine Sciences, Shahid Beheshti University of Medical Sciences, Tehran, Iran; 5 Nutrition and Endocrine Research Center, Research Institute for Endocrine Sciences, Shahid Beheshti University of Medical Sciences, Tehran, Iran

**Keywords:** Type 2 diabetes, Risk factors

## Abstract

This study aims to assess the effects of a community-based lifestyle intervention program on the incidence of type 2 diabetes (T2D). For this purpose, three communities in Tehran were chosen; one community received a face-to-face educational session embedded in a long-term community-wide lifestyle intervention aimed at supporting lifestyle changes. We followed up 9,204 participants (control: 5,739, intervention: 3,465) triennially from 1999 to 2015 (Waves 1–5). After a median follow-up of 3.5 years (wave 2), the risk of T2D was 30% lower in the intervention community as compared with two control communities by (Hazard-ratio: 0.70 [95% CI 0.53; 0.91]); however, the difference was not statistically significant in the following waves. After a median follow-up of 11.9 years (wave 5), there was a non-significant 6% reduction in the incidence of T2D in the intervention group as compared to the control group (Hazard-ratio: 0.94 [0.81, 1.08]). Moreover, after 11.9 years of follow-up, the intervention significantly improved the diet quality measured by the Dietary Approaches to Stop Hypertension concordance (DASH) score. Mean difference in DASH score in the intervention group versus control group was 0.2 [95% CI 0.1; 0.3]. In conclusion, the intervention prevented T2D by 30% in the short-term (3.5 years) but not long-term; however, effects on improvement of the diet maintained in the long-term.

**Registration:** This study is registered at IRCT, a WHO primary registry (https://irct.ir). The registration date 39 is 2008-10-29 and the IRCT registration number is IRCT138705301058N1.

## Introduction

The burden of type 2 diabetes (T2D) is high and increasing in Low- and Middle-Income Countries (LMICs) like Iran^1^. Iranians have a high risk of developing T2D with an incidence rate of the disease being 11 per 1,000 person-years^2^. Given the high burden of T2D in Iran, there is an urgent need for effective prevention programs for T2D that can be implemented nationally.

Community-wide strategies are vital to have a major impact on trends in raising the T2D epidemic^3–5^. Several studies have assessed the effects of community-wide lifestyle interventions on the prevention of T2D. For this aim, community-wide interventions were delivered to a community living within a certain geographic area and their outcomes were compared with other similar communities in distinct geographic areas^6–8^. Previous community-wide lifestyle intervention studies showed a reduction in risk factors of T2D including significant beneficial effects on poor diet habits^9–13^, obesity^9,14–17^, low physical activity^9,13,18^, and smoking^9–13,16,19–28^. However, only a few of them assessed the effect of their program on the incidence of T2D^14,29^. Moreover, none of the previous studies evaluated the maintenance of the effects of community-wide lifestyle interventions after a decade. Most community-wide lifestyle intervention studies have been conducted in high-income countries and there are only a few studies from LMICs^6–8^. Therefore, there is a pressing need for robust evidence on the long-term effects of community-wide lifestyle interventions on the incidence of T2D and related risk factors, especially in LMICs where the burden of T2D is high and increasing^1^.

The Tehran Lipid and Glucose Study (TLGS) is a long-term community-based lifestyle intervention program aimed at preventing non-communicable diseases and their risk factors^30^. Previously, we showed that the lifestyle intervention in the TLGS reduced T2D incidence and its risk factors including obesity, dyslipidemia, and dysglycemia short-term (after 3 years)^17^. The current study evaluates the long-term effectiveness of the TLGS intervention on the incidence of T2D and its risk factors.

## Methods

### Study design and participants

The TLGS was designed and implemented at two main junctures: (i) a cross-sectional study of the prevalence of NCDs and their risk factors implemented from 1999 to 2001^30^ and (ii) a prospective follow-up study along with lifestyle interventions and triennial data recollection. The protocol of TLGS has been published previously^30^. Briefly, three areas in District 13 of Tehran were selected for the study. One district was assigned to receive lifestyle intervention, and two other communities, far from the intervention area, were allocated to the control group. Of the total population living in the three areas (total population: 27,551: intervention area: 10,761, control areas: 7,858 + 8,932), 10,368 individuals who aged ≥ 20 years provided informed consent. Of these individuals, 1,164 had T2D at baseline and were excluded. As a result, 9,204 eligible participants were left for this study (5,739 participants in the control group and 3,465 participants in the intervention group). We used information of all eligible participants in the primary analysis of this study after imputing the missing values. In the sensitivity analysis, however, of the 9,204 eligible participants for this study, we excluded 2,158 participants who were absent at follow-up (23%), 178 participants with missing status for T2D in their follow-ups (2%), and 910 participants with missing values for confounders and blood glucose at baseline (10%). Eventually, the data of the remaining 5,958 participants (3,906 participants in the control group and 2052 in the intervention group) were used in the sensitivity analysis.

### Clinical and laboratory measurements

The details of clinical and laboratory measurements have been reported previously^30^. Briefly, all participants were examined at the TLGS Clinic between February 1999 and August 2001 (wave 1) and were followed up through four times: wave 2: Oct 2001–Sep 2005; wave 3: Apr 2005–Sep 2008; wave 4: Jul 2008–Oct 2011; and wave 5: Sep 2011–Jan 2015. In each wave, data on demographics, socioeconomic status, past medical history, physical activity, and smoking status were collected by completing a 110-item questionnaire. The Lipid Research Clinic (LRC) questionnaire in the baseline examination (wave 1) and the Modifiable Activity Questionnaire (MAQ) in waves 2–5 were used to assess the physical activity level^30^.

Dietary intake was collected using a validated 168-item food frequency questionnaire (FFQ)^31,32^ in waves 3–5. The FFQ was used to collect information on 17 food groups including whole grains, refined grains, dairy products, vegetables, fruits, legumes, meats, poultry, fish, nuts, seeds, dry beans, fat, oil, tea, coffee, salt, and simple sugars^31,32^. In our previous studies, we showed that the FFQ was reliable and valid for assessing the intake of different food groups in the TLGS population^31,32^. Furthermore, in another TLGS sub-study, dietary intake data of 578 randomly selected participants in the control group and the intervention group were collected at the baseline (wave 1) using the FFQ. The results showed that there was no difference between intervention and control group^33^.

Anthropometric and blood pressure measurements were taken according to standard protocols^30^. A blood sample was drawn after 12–14 h of overnight fasting to measure fasting plasma glucose (FPG), 2 h-postprandial plasma glucose (2 h-PG), high-density lipoprotein cholesterol (HDL-C), triglycerides, and total cholesterol. For the oral glucose tolerance test, 75 g anhydrous glucose was administered orally^30^. All the measurements were taken in both the intervention and control groups.

### Definition of terms

Current smoking was ascertained in participants who smoked cigarettes at least once a day or those who smoked cigarettes occasionally. Low physical activity was defined as having less than 3 days of performing sports or heavy physical activity per week in wave 1 of study (based on the LRC questionnaire) and as having less than 600 metabolic equivalents of task (MET) per week in waves 2–5 (based on MAQ questionnaire)^30^.

The quality of participants’ diets was assessed according to Dietary Approaches to Stop Hypertension (DASH) dietary plan^34^. To this end, the cut-offs defined by the DASH diet concordance score (DASH score) was used^34^.

We discarded measurements collected after contamination. Contamination occurred if a participant assigned to the intervention group moved out from the intervention community or a participant assigned to the control group moved into the intervention community in one of the follow-up waves. A total of 267, 357, and 108 participants in the intervention group moved out from the intervention community in waves 3, 4, and 5, respectively. Also, 15, 11, and 11 participants in the control group moved into the intervention community in waves 3, 4, and 5, respectively. The data collected after contamination were discarded and the data before the contamination were used as the last available follow-up.

### Outcomes

The primary outcome was the incidence of T2D. T2D was ascertained as FPG ≥ 7.0 mmol/L or 2 h-PG ≥ 11.1 mmol/L and/or taking glucose-lowering medication^35^. The event date was considered as the half-time between the first date that T2D was diagnosed and the last known disease-free date.

Secondary outcomes were the difference between the study groups in physical activity, smoking status, energy intake, quality of diet, body mass index (BMI), waist circumference (WC), FPG, 2 h-PG, triglycerides, High-Density Lipoproteins (HDL) cholesterol, and total cholesterol in the follow-up waves. The difference in change from baseline was also assessed for physical activity, smoking status, BMI, WC, FPG, 2 h-PG, triglycerides, HDL, and total cholesterol in the follow-up waves. The difference in change from baseline was not assessed for energy intake and quality of diet because these measurements were not collected at the baseline.

### Description of lifestyle intervention

Details of the lifestyle intervention have been reported previously^17,30,36–38^; briefly, the lifestyle intervention design was adapted from the North Karelia project^39,40^ and using American Heart Association guidelines. For this purpose, findings of the need assessments and Knowledge, Attitude, and Practice (KAP) studies in the TLGS communities were also used^41,42^. The lifestyle intervention was performed to prevent non-communicable diseases and improve risk factors of the subject through improving diet, increasing physical activity, and encouraging smoking cessation. The interventions had three components: family-based, school-based, and community-wide interventions. On the other hand, the control group received routine health care.

#### Family-based lifestyle intervention

The families in the intervention area were invited to receive a single face-to-face lifestyle intervention educational session between waves 1 and 2 of the study (between 1999 and 2001). The session was initiated with individual consultation and a 2-h educational class in small groups. The session included structured advice about lifestyle changes related to improving dietary patterns, increasing physical activity, and smoking cessation using slide and video presentations. All smokers were invited to take part in a motivational consult and then referred to a cessation clinic.

Residents from the intervention community received health newsletters named “Courier of Health” every three months (between 2001 and 2011). The newsletters contained information about health topics including the food pyramid guide, weight management, health hazards of smoking, smoking-cessation techniques, the importance of daily walking, and regular physical activity, and specific exercise recommendations. Moreover, they contained summarized findings of the TLGS, including the prevalence of risk factors in their community. Pamphlets and booklets on specific topics related to lifestyle management were also distributed 2–4 times per year among the residents of the intervention community. TLGS staff collected information on how many families read the pamphlets and booklets in each wave through telephone surveys. Telephone surveys showed that 50% of households had received and paid attention to the educational pamphlets and health newsletters^37^.

#### Community-wide intervention

Participants in the intervention area were also encouraged to participate in public education in 2–4 community gatherings annually for 1.5 to 3 h between 2001 and 2011. The gatherings included social events, seminars, and religious ceremonies, particularly at mosques in the holy month of Ramadan. Public events on occasions such as World Tobacco Day and World Diabetes Day were also held. Community health projects such as sports competitions, developing sports facilities in the community, providing subsidies for local gyms and pools, and health promotion advertisements (billboards) were also established in the intervention community between 2001 and 2011. More than 80% of the households participated in at least one of public gatherings for national or religious holidays between every two examinations^38^.

#### School-based intervention

A total of 12 schools across the intervention community implemented an ongoing school-based intervention that directly targeted students, parents, teachers, staff, and the school environment^36^. Briefly, since 2001, a total of 12 healthy lifestyle sessions (45 min each) were held for the 1st-grade students, followed with three sessions for students of grades 2 and 3, in each school year. Sessions included educational courses for students including “living tobacco-free” classes. The intervention also included forming the school’s “health team” by students with the aim of peer education^38^ and labelling snacks sold at the school’s shop regarding their healthiness.

For parents, three educational sessions (60 min each) were conducted for each grade in addition to a group discussion each year^36^. For teachers, 2-day seminars and 45-min class focusing on the knowledge and skills regarding healthy behaviours were held annually^36^. Moreover, smoking was prohibited for all the schoolchildren, teachers, and employees inside the school.

### Statistical analysis

Baseline characteristics were summarized using mean (± SD) values for continuous and frequencies (%) for categorical variables in the control and intervention groups. Since triglycerides had a skewed distribution, it was summarized by the median (interquartile range).

Cox proportional hazard models were fitted to compare the incidence rate of the T2D in the study groups accounting for baseline value of potential confounders (i.e., age, sex, area of residence, education level, family history of diabetes, smoking, low physical activity, WC, BMI, systolic blood pressure, diastolic blood pressure, FPG, 2 h-PG, total cholesterol, triglycerides, and HDL-C, and self-reported drug consumption for hypertension, and dyslipidemia) and clustered nature of data for families (i.e., using robust standard errors). Also, the effect of the intervention in each wave was estimated by restricting the Cox proportional hazard models to the data collected until that particular wave. For example, to estimate the effect of the intervention until the end of wave 3, we only used the data collected from waves 1–3 and discarded the data collected in waves 4 and 5.

To compare the risk factor levels between the study groups in different waves, generalized estimating equations (GEE) were fitted in a long-form dataset including data from waves 2–5^43^. In each model, the level of risk factor was defined as the outcome and the predictors were defined to be baseline level of risk factor, time-point variable (i.e., waves 2–5), interventions status (control and intervention), an interaction term between intervention status and time-point variable, and potential confounders (i.e., age, sex, area of residence, education level, family history of diabetes, smoking, low physical activity, WC, BMI, systolic blood pressure, diastolic blood pressure, FPG, 2 h-PG, total cholesterol, triglycerides, and HDL-C, and self-reported drug consumption for hypertension, and dyslipidemia). The model also accounted for the clustered nature of data due to repeated measures using an auto-regressive correlation matrix (the autoregressive process of order 1). An autoregressive correlation matrix was chosen since measurements taken further apart were less correlated than those taken closer together^44^. Logit link function for binary outcomes and identity link function for continuous outcomes were used in the GEE models.

To compare the change from baseline for the risk factor levels between the study groups in different waves, GEE models with similar link functions (as described above) and autoregressive correlation matrix were fitted in a long-form dataset including data from waves 1–5^43^. In each model, the level of risk factor was defined as the outcome and the predictors were defined to be time-point variable (i.e., waves 1–5), intervention status (control and intervention), the interaction term between intervention status and time-point variable, and potential confounders (as listed above). The estimated coefficient for interaction terms between intervention status and time-point variable were used to compare study groups in change in risk factor levels from baseline to different waves of study^43^.

In the primary analysis for primary outcome (incidence of T2D), we imputed the baseline missing values of BMI (n = 230), WC (n = 269), family history of T2D (n = 394), low-physical activity (n = 163), education level (n = 10), smoking status (n = 158), systolic/diastolic blood pressure (n = 209), FPG (n = 299), 2 h-PG (n = 880), total cholesterol (n = 297), triglycerides (n = 300), HDL (n = 307)m T2D status at the end of follow-up (n = 2,270), and time-to-event (n = 2,305). Ice package in Stata was used to produce 10 imputed datasets using linear regression models for imputing continuous variables, logistic regression for binary variables, and ordinal regression models for ordinal variables^45–47^. We imputed the time-to-event variable as described in ice package documentations and previous studies^45–47^. To analyze the secondary outcomes, we also imputed data of risk factors in follow-up waves (waves 2–5) using similar methods^45–47^. For each analysis, estimates from imputed datasets were combined using Rubin’s rule^45–47^. In the complete-case analysis, similar models were fitted in those with complete data (n = 5,958).

To account for multiple comparisons in the secondary aim of the study, the corrected p-value threshold was calculated and reported using the Bonferroni^48^ formula. Estimates with p-values less than Bonferroni threshold were considered as statistically significant. Analyses were performed using the Stata statistical software (version 14 SE).

### Research ethics

The ethics committee of the Research Institute for Endocrine Sciences, Shahid Beheshti University of Medical Sciences confirmed the design of the TLGS study. Methods were carried out following the relevant guidelines and regulations. All participants provided written informed consent.

## Results

Of 9,204 included participants, 5,285 ones were female (57.4%) with mean (± SD) age and BMI of 41.1 ± 14.52 years and 26.5 ± 4.7 kg/m^2^, respectively (Table 1). As compared to participants in the intervention group, participants in the control group had significantly higher physical activity (25% vs. 22%), education, smoking prevalence (14% vs. 11%), and FPG (4.99 vs. 4.96 mmol/L) and lower WC (86.8 vs. 87.4 cm) at baseline (Table 1).

**Table 1:** Comparison between baseline characteristics by study group; Tehran Lipid and Glucose Study 1999–2015.

Characteristics	Level	Control (n = 5,739)	Intervention (n = 3,465)	p-value
Age (year)		40.85 (14.30) (n = 5,739)	41.43 (14.88) (n = 3,465)	0.060
Sex (%)	Male	2,463 (42.9%)	1,456 (42.0%)	0.40
	Female	3,276 (57.1%)	2,009 (58.0%)	
	Missing	0 (0.0%)	0 (0.0%)	
Family history of type 2 diabetes	No	4,057 (70.7%)	2,448 (70.6%)	0.57
	Yes	1,422 (24.8%)	883 (25.5%)	
	Missing	260 (4.5%)	134 (3.9%)	
Education (%)	Less than 6 years	1,757 (30.6%)	1,212 (35.0%)	< 0.001
	6–12 years	3,198 (55.7%)	1,837 (53.0%)	
	Post-school education	776 (13.5%)	414 (11.9%)	
	Missing	8 (0.1%)	2 (0.1%)	
Low physically active (%)	No	1,438 (25.1%)	769 (22.2%)	0.001
	Yes	4,188 (73.0%)	2,646 (76.4%)	
	Missing	113 (2.0%)	50 (1.4%)	
Current smoking (%)	No	4,802 (83.7%)	2,979 (86.0%)	0.013
	Yes	827 (14.4%)	438 (12.6%)	
	Missing	110 (1.9%)	48 (1.4%)	
Weight (kg)		70.03 (13.06) (n = 5,584)	69.79 (13.08) (n = 3,390)	0.39
Height (cm)		162.74 (9.21) (n = 5,600)	162.05 (9.06) (n = 3,408)	< 0.001
Body mass index (kg/m2)		26.48 (4.71) (n = 5,584)	26.59 (4.69) (n = 3,390)	0.28
Waist circumference (cm)		86.81 (12.11) (n = 5,564)	87.36 (12.10) (n = 3,371)	0.036
Systolic blood pressure (mm Hg)		117.75 (17.61) (n = 5,593)	118.25 (18.31) (n = 3,402)	0.20
Diastolic blood pressure (mm Hg)		77.30 (10.47) (n = 5,593)	77.14 (10.82) (n = 3,402)	0.47
Fasting plasma glucose (mmol/L)		4.99 (0.54) (n = 5,554)	4.96 (0.55) (n = 3,351)	0.024
Postprandial plasma glucose (mmol/L)		5.92 (1.65) (n = 5,194)	5.93 (1.63) (n = 3,130)	0.77
Total cholesterol (mmol/L)		5.32 (1.18) (n = 5,555)	5.33 (1.15) (n = 3,352)	0.62
HDL-C (mmol/L)		1.10 (0.28) (n = 5,550)	1.09 (0.28) (n = 3,347)	0.68
Triglycerides (mmol/L)		1.54 (1.05, 2.24) (n = 5,554)	1.54 (1.03, 2.26) (n = 3,350)	0.67

Baseline characteristics were summarized using mean (standard deviation: SD) values for continuous and frequencies (%) for categorical variables. Since triglycerides had a skewed distribution, it was summarized by the median (interquartile range). Baseline characteristics were compared between study groups using student’s *T*-test, chi-square test, and Mann–Whitney *U* test, whichever appropriate.

After a median follow-up of 3.5 years (IQR 2.7–4.2) (wave 2), 4.5% (95% CI 3.7%; 5.2%) of participants in the control group and 3.8% (95% CI 3.1%; 4.5%) participants in the intervention group developed T2D. It resulted in incidence rates of 14.4 and 9.2 per 1,000 person-years in the control and the intervention groups, respectively. The hazard of incident T2D was significantly 30% lower in the intervention group compared to the control group (IRR 0.70 [0.53; 0.91], p-value: 0.009). This difference, however, disappeared in the following waves (Table 2). After a median follow-up of 11.9 years (IQR 6.6–13.3) (wave 5), 14.1% (95% CI 13.2%; 15.1%) of participants in the control group and 11.1% (95% CI 10.1%; 12.6%) of participants in the intervention group were diagnosed with T2D with an incidence rate of 12.9 and 11.9 per 1,000 person-years in the control and intervention groups, respectively. There was a non-significant 6% reduction in the incidence rate of T2D in the intervention group compared to the control group [Hazard ratio: 0.94 (0.81, 1.08)]. Findings from complete case analyses were similar to those from the primary analysis (Table 2).

**Table 2:** The incidence rate of T2D by study group; the Tehran Lipid and Glucose Study 1999–2015.

Primary analysis			
	Follow-up (years), median (IQR)	Hazard ratio (95% CI)	p-value
Wave 2	3.5 [2.7; 4.2]	0.70 [0.53; 0.91]	0.009
Wave 3	6.0 [5.2; 7.1]	0.96 [0.79; 1.18]	0.718
Wave 4	9.0 [6.8; 10.2]	0.94 [0.80; 1.11]	0.468
Wave 5	11.9 [6.6; 13.3]	0.94 [0.81; 1.08]	0.385

Complete case analysis			
	Follow-up (years), median (IQR)	Hazard ratio (95% CI)	p-value
Wave 2	3.4 [2.7; 4.2]	0.70 [0.51; 0.97]	0.034
Wave 3	6.0 [5.3; 7.1]	0.97 [0.77; 1.24]	0.830
Wave 4	9.1 [7.1; 10.2]	0.95 [0.79; 1.16]	0.641
Wave 5	12.0 [7.8; 13.3]	0.94 [0.80; 1.12]	0.505

In the primary analysis, we imputed the baseline missing values of BMI (n = 230), WC (n = 269), family history of type 2 diabetes (n = 394), low-physical activity (n = 163), education level (n = 10), smoking status (n = 158), systolic/diastolic blood pressure (n = 209), FPG (n = 299), 2 h-PG (n = 880), total cholesterol (n = 297), triglycerides (n = 300), HDL (n = 307), all-wave follow-up time (n = 2,305), and T2D status (n = 2,270). Ice package in Stata was used to produce 10 imputed datasets using linear regression models for imputing continuous variables, logistic regression for binary variables, and ordinal regression models for ordinal variables. We imputed the time-to-event variable as described in ice package documentations and previous studies. Moreover, we used age, sex, drug consumption for dyslipidemia, and hypertension as axillary variables in the imputation process. In the complete-case analysis, similar models were fitted in those with complete data (n = 5,958).

Cox proportional hazard models were fitted to compare the incidence rate of the T2D in the study groups accounting for baseline value of potential confounders (i.e., age, sex, area of residence, education level, family history of diabetes, smoking, low physical activity, WC, BMI, systolic blood pressure, diastolic blood pressure, FPG, 2 h-PG, total cholesterol, triglycerides, and HDL-C, and self-reported drug consumption for hypertension, and dyslipidemia) and clustered nature of data for families (i.e., using robust standard errors). Moreover, the effect of the intervention in each wave was estimated by restricting the Cox proportional hazard models to the data collected until that particular wave (e.g., to estimate the effect of the intervention until the end of wave 3), we only used the data collected from waves 1–3 and discarded the data collected in waves 4 and 5.

Table 3 compares the change in the lifestyle risk factors of T2D. Based on the Bonferroni method, the corrected p-value threshold was 0.00058 for the secondary aim of the study. Adjusting for the differences between study groups at baseline, at wave 2 of TLGS, the intervention group indicates a significantly lower FPG (by 0.1 mmol/L; p-value < 0.0001) and a significantly higher HDL (by 0.03 mmol/L; p-value < 0.0001). Similar findings were observed when comparing the changes from baseline to wave 2 for each risk factor. In the final wave (wave 5), the intervention group had significantly higher levels of DASH score (0.2; p-value < 0.0001). Generally, similar results were observed in complete case analysis (data not shown).

**Table 3:** Comparison between study groups in the level of T2D risk factors in each wave as well as their change from baseline.

Risk factor	Wave 2		Wave 3		Wave 4		Wave 5	
	Mean (SD)	Change from baseline	Mean (SD)	Change from baseline	Mean (SD)	Change from baseline	Mean (SD)	Change from baseline
**Waist**								
Intervention	91.5 (12.0)	4.2 (3.9, 4.5)	92.7 (12.4)	5.4 (5.1, 5.8)	96.2 (11.8)	8.9 (8.6, 9.3)	96.1 (11.8)	8.8 (8.4, 9.3)
Control	91.6 (12.1)	4.9 (4.6, 5.1)	92.3 (12.3)	5.6 (5.4, 5.8)	96.2 (11.8)	9.4 (9.2, 9.7)	96.3 (11.8)	9.5 (9.2, 9.8)
Between group difference	− 0.5 (− 0.8, − 0.1) p-value = 0.021	− **0.6 (**− **1.0, **− **0.3)** **p-value < 0.0001**	0.0 (− 0.3, 0.4) p-value = 0.875	− 0.2 (− 0.6, 0.2) p-value = 0.434	− 0.3 (− 0.7, 0.0) p-value = 0.065	− 0.5 (− 0.9, − 0.1) p-value = 0.017	− 0.5 (− 0.9, − 0.1) p-value = 0.011	− 0.7 (− 1.1, − 0.2) p-value = 0.004
**Body mass index**								
Intervention	27.6 (4.8)	1.0 (0.8, 1.2)	28.0 (4.8)	1.4 (1.2, 1.6)	28.5 (6.7)	1.9 (1.7, 2.2)	28.7 (5.1)	2.1 (1.9, 2.4)
Control	27.5 (4.8)	1.0 (0.9, 1.2)	27.9 (4.8)	1.4 (1.3, 1.6)	28.6 (9.9)	2.1 (2.0, 2.3)	28.8 (5.3)	2.3 (2.1, 2.5)
Between group difference	0.0 (− 0.2, 0.2) p-value = 0.957	0.0 (− 0.2, 0.2) p-value = 0.821	− 0.1 (− 0.3, 0.1) p-value = 0.504	− 0.1 (− 0.3, 0.2) p-value = 0.509	− 0.2 (− 0.5, 0.1) p-value = 0.219	− 0.2 (− 0.5, 0.1) p-value = 0.251	− 0.1 (− 0.3, 0.1) p-value = 0.190	− 0.2 (− 0.4, 0.1) p-value = 0.273
**Fasting plasma glucose**								
Intervention	4.99 (0.76)	0.03 (− 0.01, 0.06)	5.13 (0.97)	0.17 (0.13, 0.21)	5.49 (1.14)	0.53 (0.48, 0.57)	5.60 (1.38)	0.64 (0.59, 0.69)
Control	5.13 (0.72)	0.14 (0.11, 0.17)	5.09 (0.88)	0.11 (0.07, 0.14)	5.47 (1.20)	0.49 (0.45, 0.52)	5.60 (1.44)	0.61 (0.57, 0.65)
Between group difference	− **0.13 (**− **0.17, **− **0.08)** **p-value < 0.0001**	− **0.11 (**− **0.16, **− **0.07)** **p-value < 0.0001**	0.05 (0.00, 0.09) p-value = 0.035	0.06 (0.01, 0.11) p-value = 0.019	0.03 (− 0.02, 0.08) p-value = 0.211	0.04 (− 0.01, 0.10) p-value = 0.133	0.02 (− 0.04, 0.08) p-value = 0.539	0.03 (− 0.04, 0.10) p-value = 0.358
**Post**-**prandial glucose**								
Intervention	6.1 (2.2)	0.2 (0.1, 0.3)	6.2 (2.6)	0.3 (0.2, 0.4)	6.7 (3.0)	0.8 (0.7, 0.9)	7.2 (3.6)	1.3 (1.2, 1.4)
Control	6.3 (2.2)	0.4 (0.3, 0.4)	6.2 (2.5)	0.3 (0.2, 0.4)	6.6 (3.0)	0.8 (0.7, 0.8)	7.2 (3.6)	1.4 (1.2, 1.5)
Between group difference	− 0.1 (− 0.3, 0.0) p-value = 0.021	− 0.1 (− 0.3, 0.0) p-value = 0.009	0.0 (− 0.1, 0.1) p-value = 0.800	0.0 (− 0.1, 0.1) p-value = 0.751	0.0 (− 0.1, 0.2) p-value = 0.720	0.0 (− 0.1, 0.2) p-value = 0.815	− 0.1 (− 0.2, 0.1) p-value = 0.496	− 0.1 (− 0.2, 0.1) p-value = 0.513
**Triglycerides**								
Intervention	1.74 (1.10)	− 0.09 (− 0.12, − 0.05)	1.73 (1.08)	− 0.10 (− 0.14, − 0.06)	1.68 (1.05)	− 0.15 (− 0.20, − 0.11)	1.71 (1.01)	− 0.11 (− 0.16, − 0.07)
Control	1.80 (1.20)	− 0.02 (− 0.05, 0.01)	1.78 (1.09)	− 0.04 (− 0.08, − 0.01)	1.73 (1.12)	− 0.10 (− 0.13, − 0.06)	1.73 (1.01)	− 0.10 (− 0.14, − 0.06)
Between group difference	− 0.06 (− 0.11, − 0.01) p-value = 0.012	− 0.07 (− 0.11, − 0.02) p-value = 0.007	− 0.05 (− 0.09, − 0.01) p-value = 0.021	− 0.06 (− 0.11, 0.00) p-value = 0.037	− 0.05 (− 0.09, − 0.01) p-value = 0.025	− 0.05 (− 0.11, 0.00) p-value = 0.061	− 0.01 (− 0.05, 0.03) p-value = 0.629	− 0.02 (− 0.07, 0.04) p-value = 0.581
**HDL**-**cholesterol**								
Intervention	1.02 (0.27)	− 0.08 (− 0.09, − 0.07)	1.11 (0.27)	0.02 (0.01, 0.03)	1.22 (0.29)	0.13 (0.11, 0.14)	1.28 (0.32)	0.19 (0.18, 0.20)
Control	0.99 (0.26)	− 0.10 (− 0.11, − 0.10)	1.07 (0.27)	− 0.03 (− 0.04, − 0.02)	1.23 (0.29)	0.13 (0.12, 0.14)	1.26 (0.32)	0.17 (0.16, 0.18)
Between group difference	**0.03 (0.01, 0.04)** **p-value < 0.0001**	**0.03 (0.02, 0.04)** **p-value < 0.0001**	**0.04 (0.03, 0.06)** **p-value < 0.0001**	**0.05 (0.03, 0.06)** **p-value < 0.0001**	− 0.01 (− 0.02, 0.00) p-value = 0.173	− 0.01 (− 0.03, 0.01) p-value = 0.375	0.02 (0.01, 0.03) p-value = 0.001	0.02 (0.00, 0.03) p-value = 0.013
**Total cholesterol**								
Intervention	4.9 (1.0)	− 0.4 (− 0.4, − 0.3)	5.0 (1.0)	− 0.3 (− 0.3, − 0.3)	5.0 (1.0)	− 0.3 (− 0.4, − 0.3)	5.1 (1.0)	− 0.2 (− 0.2, − 0.1)
Control	5.0 (1.1)	− 0.3 (− 0.3, − 0.3)	5.0 (1.0)	− 0.3 (− 0.4, − 0.3)	5.1 (1.0)	− 0.2 (− 0.3, − 0.2)	5.1 (1.0)	− 0.2 (− 0.2, − 0.2)
Between group difference	− 0.1 (− 0.1, 0.0) p-value = 0.012	− 0.1 (− 0.1, 0.0) p-value = 0.010	0.0 (0.0, 0.1) p-value = 0.053	0.0 (0.0, 0.1) p-value = 0.170	− 0.1 (− 0.1, 0.0) p-value = 0.004	− 0.1 (− 0.1, 0.0) p-value = 0.015	0.0 (0.0, 0.1) p-value = 0.238	0.0 (0.0, 0.1) p-value = 0.499
**DASH score**								
Intervention	NA	NA	5.9 (1.5)	NA	6.8 (1.4)	NA	6.9 (1.4)	NA
Control	NA	NA	5.8 (1.5)	NA	6.6 (1.5)	NA	6.7 (1.5)	NA
Between group difference	NA	NA	0.1 (0.0, 0.3) p-value = 0.091	NA	**0.2 (0.1, 0.3)** **p-value = 0.0001**	NA	**0.2 (0.1, 0.3)** **p-value = 0.0003**	NA
**Energy intake**								
Intervention	NA	NA	2,347 (1,041)	NA	2,481 (1,025)	NA	2,432 (1,020)	NA
Control	NA	NA	2,371 (1,066)	NA	2,490 (1,053)	NA	2,366 (1,007)	NA
Between group difference	NA	NA	− 17 (− 106, 72) p-value = 0.690	NA	− 2 (− 72, 68) p-value = 0.946	NA	73 (16, 131) p-value = 0.014	NA
	N (%)	Change from baseline (Odds ratio)	N (%)	Change from baseline (Odds ratio)	N (%)	Change from baseline (Odds ratio)	N (%)	Change from baseline (Odds ratio)
**Smoking**								
Intervention	424 (12.2)	0.9 (0.8, 1.1)	376 (10.9)	0.8 (0.7, 0.9)	379 (10.9)	0.8 (0.7, 1.0)	384 (11.1)	0.8 (0.7, 1.0)
Control	878 (15.3)	1.1 (1.0, 1.1)	832 (14.5)	1.0 (0.9, 1.1)	783 (13.6)	0.9 (0.8, 1.0)	768 (13.4)	0.9 (0.8, 1.0)
Between group difference	0.72 (0.56, 0.91) p-value = 0.008	0.88 (0.76, 1.02) p-value = 0.085	**0.62 (0.48, 0.79)** **p-value < 0.001**	0.81 (0.69, 0.96) p-value = 0.014	0.74 (0.57, 0.96) p-value = 0.024	0.89 (0.74, 1.08) p-value = 0.246	0.79 (0.61, 1.04) p-value = 0.092	0.93 (0.76, 1.14) p-value = 0.496
**Low physical activity**								
Intervention	424 (12.2)	0.3 (0.2, 0.3)	376 (10.9)	0.2 (0.2, 0.2)	379 (10.9)	0.2 (0.2, 0.2)	384 (11.1)	0.2 (0.2, 0.2)
Control	878 (15.3)	0.3 (0.3, 0.3)	832 (14.5)	0.2 (0.2, 0.2)	783 (13.6)	0.2 (0.2, 0.3)	768 (13.4)	0.2 (0.2, 0.2)
Between group difference	1.00 (0.84, 1.19) p-value = 0.974	0.86 (0.71, 1.04) p-value = 0.113	1.10 (0.98, 1.23) p-value = 0.118	0.94 (0.81, 1.09) p-value = 0.436	0.92 (0.82, 1.03) p-value = 0.135	0.79 (0.68, 0.92) p-value = 0.002	0.99 (0.86, 1.14) p-value = 0.886	0.85 (0.72, 1.01) p-value = 0.064

To compare the risk factor levels between the study groups in different waves, generalized estimating equations (GEE) were fitted in a long-form dataset including data from waves 2–5. In each model, the level of risk factor was defined as the outcome and the predictors were defined to be baseline level of risk factor, time-point variable (i.e., waves 2–5), interventions status (control and intervention), the interaction term between intervention status and time-point variable, and potential confounders (i.e., age, sex, area of residence, education level, family history of diabetes, smoking, low physical activity, WC, BMI, systolic blood pressure, diastolic blood pressure, FPG, 2 h-PG, total cholesterol, triglycerides, and HDL-C, and self-reported drug consumption for hypertension, and dyslipidemia). The model also accounted for the clustered nature of data due to repeated measurement using an auto-regressive correlation matrix (the autoregressive process of order 1). The autoregressive correlation matrix was chosen since measurements taken further apart were less correlated than those taken closer together. Logit link function for binary outcomes and identity link function for continuous outcomes were used in the GEE models.

To compare change from baseline for the risk factor levels between the study groups in different waves, GEE models with similar link functions (as described above) and autoregressive correlation matrix were fitted in a long-form dataset including data from waves 1–5. In each model, the level of risk factor was defined as the outcome and the predictors were defined to be time-point variable (i.e., waves 1–5), intervention status (control and intervention), the interaction term between intervention status and time-point variable, and potential confounders (as listed above). The estimated coefficient for interaction terms between intervention status and time-point variable were used to compare study groups in change in risk factor levels from baseline to different waves of the study.

Based on the Bonferroni method, the corrected p-value threshold was 0.00058 for the secondary aim of the study.

## Discussion

This research is one of the few studies reporting the long-term effects of a community-wide lifestyle intervention on T2D prevention in an LMIC. Although the intervention reduced the risk of T2D by 30% short-term (wave 2: 3.5 years of follow-up), this effect was not maintained until the end of the study (wave 5) with a non-significant 6% reduction in the risk of T2D after 11.9 years of follow-up. Nevertheless, the effect of lifestyle intervention on the improvement of diet quality maintained after 11.9 years.

Our findings are in line with those of a recent study in the TLGS that showed that lifestyle intervention prevented metabolic syndrome short-term (waves 2 and 3). However, this effect was not maintained in the long run (wave 4 and wave 5)^37^. Moreover, our recent study in the adolescent participants of the TLGS, showed that the lifestyle intervention prevented metabolic syndrome in a short time (wave 2) but not in long-term (wave 4)^36^. There is limited evidence about the long-term effects of a community-wide lifestyle intervention program on the incidence of T2D or the prevalence of its risk factors. Repeated surveys in the Isfahan Healthy Heart Program, a multi-component community-wide lifestyle intervention, showed a non-significant (0.8%) reduction in the prevalence of T2D in the intervention group compared to the controls after 7 years of follow-up^29^. Similarly, studies conducted in high-income countries showed no significant reduction in the incidence of T2D in the long-term^14,49,50^. Regarding changes in the risk factors of T2D, several studies showed that the community-wide lifestyle intervention programs had significant long-term effects on the reduction of T2D risk factors^6–8,51^. In a community-wide lifestyle intervention in the Coalfields district of New South Wales, Australia^52^, a significant reduction in poor dietary habits and BMI was observed after 10 years of follow-up.

Our research demonstrates that a low-cost and pragmatic intervention consisting of a face-to-face educational session embedded in a community-wide intervention can reduce the incidence of T2D short-term and improve the quality of diet in the long-term. Therefore, if scaled up, this program can prevent a considerable number of new cases of T2D as well as other non-communicable diseases in Iran. However, we also showed that the intervention was not effective in the long term. Underscoring this issue needs more attention to ensure lifestyle intervention effectiveness in the long-term. There is limited evidence regarding the factors contributing to the maintenance of long-term success of community-wide lifestyle intervention programs especially beyond a period of 24 months^53^. Previous studies suggested that the lack of maintenance in the effects of lifestyle interventions may be due to the inability of participants in maintaining healthy eating and exercise behaviours over time^53,54^ and long-term maintenance may increase by removing the barriers in lifestyle modification^55^. Based on previous studies^56–61^, the most important barriers to healthy nutrition and physical activity were interpersonal/cultural effects, lack of access to healthy foods, food preferences, media advertisements, nutrition transition, lack of time, motivation, and prioritizing other activities over sports and high costs of the facilities. Moreover, a booster face-to-face educational session every 3 years might help maintain the effectiveness of the lifestyle intervention for a longer period.

This study has several strengths including a large sample size and more than a decade-long follow-up. Moreover, the team and the methods for data collection had minimal changes throughout the study. This is one of the few community trials that has collected and compared the dietary patterns in detail between study groups. The findings of this study, however, should be interpreted in light of several limitations of which, such as the non-randomized design of the study. As another limitation, 35% of participants had at least one missing value in their variables of interest. Moreover, TLGS did not collect data on T2D knowledge. An increase in T2D knowledge is one of the main goals of community-wide lifestyle intervention programs for T2D prevention as it can result in the gradual development of healthy beliefs/attitudes and a meaningful change in behaviour. Lack of T2D knowledge data averted further investigations on potential causes for lack of long-term effectiveness. For example, T2D knowledge data could have helped distinguish whether the lack of long-term effectiveness is mainly due to external and internal barriers that prevent participants to apply their knowledge in practice or it was mainly due to a decrease in T2D knowledge of TLGS participants.

In conclusion, the lifestyle intervention reduced the risk of T2D by 30% after 3.5 years of follow-up (wave 2 of study), a change that was not maintained in the following waves with a non-significant 6% reduction in T2D risk after 11.9 years of follow-up. Nevertheless, the effect of lifestyle intervention on the quality of diet maintained even after 11.9 years of follow-up.

## Data Availability

Data are available upon request.
